# The Antimicrobial Peptide LL-37 as a Predictor Biomarker for Periodontitis with the Presence and Absence of Smoking: A Case-Control Study

**DOI:** 10.1155/2023/5581267

**Published:** 2023-09-06

**Authors:** Wael Abdulazeez Kzar, Raghad Fadhil Abbas, Hashim Mueen Hussein

**Affiliations:** ^1^Department of Periodontology, College of Dentistry, University of Baghdad, Baghdad, Iraq; ^2^Department of Conservative Dentistry, College of Dentistry, Mustansiriyah University, Baghdad, Iraq

## Abstract

**Background:**

A major issue is finding a valid biomarker able to diagnose periodontal disease with the presence and absence of risk factors. Indeed, the association between smoking and periodontal diseases and its impact on the manifestation of antimicrobial peptides has been delineated in clinical and epidemiological investigations. The antimicrobial peptide cathelicidin (LL-37) is pivotal in preserving periodontal health.

**Objectives:**

This investigation examines and contrasts the levels of cathelicidin in the saliva of smokers and nonsmokers of periodontitis. The study also seeks to establish this biomarker's diagnostic ability to differentiate between periodontal health and disease.

**Materials and Methods:**

The study involved the collection of unstimulated saliva samples from 160 participants, comprising 80 patients diagnosed with periodontitis (40 of whom were smokers and 40 were nonsmokers) and 80 periodontitis-free individuals (40 smokers and 40 nonsmokers). The clinical periodontal parameters were assessed, including recording the probing pocket depth, the level of clinical attachment, and the percentage of bleeding on probing. Subsequently, enzyme-linked immunosorbent assays were conducted to quantify the protein levels of LL-37 in the saliva samples obtained from the subjects mentioned above.

**Results:**

The highest level of salivary LL-37 was found in the nonsmoker periodontitis (NSP) patients, followed by the group of smoker periodontitis (SP) and then nonsmoker healthy (NSH) group, while the lowest level was found in the healthy smoker (HS) group. At the same time, the LL-37 seems to be a very good biomarker in differentiating periodontal health from disease with the presence and absence of smoking.

**Conclusion:**

Periodontitis results in a significant elevation of salivary LL-37 levels in smoker and nonsmoker patients compared to healthy individuals. These levels are positively correlated with the periodontal parameter and can serve as a valuable diagnostic tool to predict periodontitis, whereas smoking significantly reduces these levels.

## 1. Introduction

Periodontitis is a pathological state characterized by inflammation of the periodontium, which is caused by the presence of various microorganisms. The development and advancement of periodontitis are impacted by the host's susceptibility and its interaction with the microbiota of the biofilm [1–3]. One of the pivotal antimicrobial peptides (AMPS) involved in the innate immune response against periodontitis is cathelicidin (LL-37), with a critical function to perform. This peptide is a diminutive, positively charged amphipathic peptide with antimicrobial properties. The synthesis of cathelicidin occurs in various types of cells, including epithelial cells and cells involved in the immune response, such as neutrophils, lymphocytes, natural killer cells, monocytes, and macrophages. The peptide displays various pleiotropic characteristics such as antimicrobial, chemotactic, and immunomodulatory functions [4, 5]. Several studies provided evidence of a direct association between the degree of periodontal tissue destruction and the increase in LL-37 expression [6–8].

The periodontium experiences an increase in host response due to the invasion of microbes. Consequently, there has been a notable increase in the production of proinflammatory mediators, including interleukins, tumor necrosis factor-*α*, matrix metalloproteinases (MMPs), and prostaglandin E2 [9, 10]. The increase in the number and activation of neutrophils in periodontitis results in an elevated level of LL-37, as neutrophile is the primary source of this peptide [11–13] since salivary LL-37 is derived from neutrophils that infiltrate the oral cavity via the junctional epithelium at sites of inflammation with further sources including both epithelial cells of the gingiva and the mucosa [14–16].

The progression of periodontal degradation in conjunction with elevated levels of LL-37 is believed to be an inherent immune reaction to periodontopathic bacteria, serving as a protective measure in periodontal tissue, as claimed by Takeuchi et al. [17]. Also, the elevated level of salivary LL-37 could be attributed to the inflammatory reaction in the periodontium, which significantly reduces the oxygen content and inhibits the neutrophile's oxygen-mediated killing. This situation calls for the production of LL-37 by neutrophils as a part of the nonoxidative antimicrobial mechanism in which LL-37 is released into tissues during inflammation [14, 18].

Smoking is widely recognized as a significant risk factor for the development of chronic noncommunicable diseases. Tobacco-related diseases have emerged as a prominent contributor to global mortality rates [19, 20]. The negative impact of cigarette smoking on the occurrence and advancement of periodontitis has been well documented [21]. Studies have demonstrated that smoking is associated with increased susceptibility to periodontal disease, with a probability of up to sevenfold higher than individuals who do not smoke [22–25]. Moreover, research indicates that smoker individuals exhibit more severe periodontal destruction than nonsmokers [26–31]. This relationship is dose-dependent, with the severity of destruction being linked to the number of cigarettes smoked and the duration of smoking [30, 32–34].

Currently, clinicians employ radiographic evidence of bone loss and clinical periodontal parameters as diagnostic tools to identify and assess the severity of periodontal disease. Nevertheless, significant constraints in utilizing clinical measurements have prompted the exploration of alternative methods for diagnosing and monitoring periodontal disease [27]. Hence, the discovery of patient-specific biological markers that can indicate the rate of risk, track the progression of diseases, assess the state of health, and predict treatment outcomes would provide substantial clinical benefits. Saliva is gaining prominence as a widely utilized diagnostic fluid owing to its bioavailability, cost-effectiveness, noninvasive accessibility, collection standards that are minimally sensitive to technique, and the relative stability of salivary analytes during storage [35].

Considering the observations mentioned above in mind, it is imperative to acknowledge the impacts of smoking on the expression levels of antimicrobial peptide levels in saliva, given the deleterious effects of smoking on the human body and the crucial role of these peptides in the immune system, and study a valid biomarker to diagnose periodontitis regardless of the presence or absence of smoking. Accordingly, the current study is aimed at determining whether there were variations in LL-37 concentration for periodontitis in smoker and nonsmoker patients. Then, we resolved to evaluate the diagnostic accuracy of recognized salivary LL-37 compared to gold standard clinical periodontal parameters, probing pocket depth (PPD) and clinical attachment loss (CAL), in differentiating periodontitis from periodontal health.

## 2. Methods and Materials

### 2.1. Study Design

The present study is an observational case-control investigation carried out at the Department of Periodontics, College of Dentistry, University of Thiqar, Iraq. From February 2022 to October 2022, a study was conducted involving the voluntary enrollment of individuals diagnosed with periodontitis and healthy individuals who were both smokers and nonsmokers. The current investigation was conducted in adherence to the ethical guidelines delineated in the Declaration of Helsinki by the World Medical Association (1964). Furthermore, it was approved by the ethical committee of the College of Dentistry, University of Baghdad (reference number: 500 on 19 January 2022).

### 2.2. Study Population

The study population comprised 160 determined according to a previously done pilot study to calculate the exact sample size involved in the present investigation to avoid attrition bias. The pilot study was done by taking ten samples for each group, and the ELISA examination was done to evaluate the concentration of LL-37 in saliva. The exact sample size was then calculated according to the formula mentioned by Sharma et al. in 2020 [36] for a case-control study. “Sample size = *r* + 1/*r* × (SD)2 × (*Zβ* + *Zα*/2)2/*d*2.”

Before conducting the periodontal examination, the medical and dental history was obtained and carefully reviewed along with the exclusion criteria. Moreover, each participant involved in the current investigation was requested to sign an informed consent form after providing all the evidence describing the purpose of the investigation. Subsequently, the selected subjects were systemically healthy other than the case definition mentioned regarding periodontitis. The involved participants were classified into distinct groups based on their periodontal clinical status:

1. NSH group: nonsmokers with clinically healthy periodontium
2. SH group: smokers with clinically healthy periodontium
3. NSP group: smoker patients with periodontitis
4. SP group: smoker patients with periodontitis

The subjects encompassed in the existing study were the control group with healthy periodontium exhibiting a PPD of ≤3 mm, BOP of ≤10%, and intact periodontium, while the periodontitis groups were defined as the presence of interdental CAL at ≥2 nonadjacent teeth or buccal or oral CAL ≥ 3 mm with the presence of pocket depth > 3 mm, which is detectable at ≥2 teeth [26]. Besides, all periodontitis cases exhibited a generalized form (≥30% of teeth involved) and unstable status (PPD ≥ 5 mm or PPD 4 mm with BOP) [37]. A calibrated examiner carried out the dental examination. The procedure consisted of recording the periodontal parameters, including the pocket depth, clinical attachment loss, and assessment of bleeding on probing.

Additionally, the smoking status criteria utilized in the present study were based on the Centers for Disease Control and Prevention (CDC) guidelines. Specifically, individuals who had smoked more than 16 cigarettes per day within the previous 30 days were classified as heavy smokers [38].

### 2.3. Eligibility Criteria

The inclusion criteria for the study comprised patients who were systemically healthy except for the case definition criteria and meet the minimum requirement of having 20 teeth. In addition, patients should have no evidence of any systemic disease or have taken any antibiotics within the past three months. Conversely, the exclusion criteria for this study encompassed patients who had medical conditions such as diabetes, immunologic diseases, and hepatitis, as well as those who were receiving antibiotics or periodontal therapy within the preceding six months. Patients with systemic conditions like liver or kidney dysfunction, inflammatory bowel disease, i.e., Crohn's disease, and former organ transplantation or malignancy history were also excluded from the study.

Further exclusion criteria included any previous extensive periodontal procedure or ongoing active periodontal treatment, patients wearing orthodontic appliances, pregnant females, and patients with any oral lesion unrelated to periodontitis, e.g., aphthous ulcer and lichen planus.

### 2.4. Collection of Salivary Sample

Following the measurement of the periodontal parameters, samples of saliva were obtained after 1 hour to ensure clearance of the mouth from any bleeding pockets or sulcus. Before saliva collection, all participants received instructions to refrain from consuming any food or beverage, except for water, for one hour. The participant was then instructed to sit relaxed and allow saliva to roll. A universal collector was used, and the patients were asked to keep their heads slightly forward and tilted to facilitate the salivary flow without any stimulus until a volume of 5 ml was obtained. The collected samples were centrifuged at a rate of 4500 revolutions per minute for 30 minutes and then stored at −20°C until analysis.

### 2.5. Statistical Analysis

GraphPad Prism (v. 9.0) software was used for data description, analysis, and presentation. Results were expressed as mean, standard deviation, and percentage. The distribution of the data was checked by using the Shapiro-Wilk normality test. Multiple group comparisons were conducted using ANOVA followed by Tukey's post hoc analysis and an unpaired *t*-test to compare the two groups. The receiver operating characteristic (ROC) curve and area under the curve (AUC) were used to determine the sensitivity and specificity of the biomarker. Pearson's correlation test performed the correlation between concentrations of salivary LL-37 with the clinical parameters of the patients. All the statistical analyses were significant at *P* value < 0.05. Finally, the interpretation of biomarker analysis results and statistics done by a blinded investigator was not influenced by the knowledge of the clinical measurement results to avoid bias.

## 3. Results

After screening 260 subjects for their eligibility criteria, only 160 were involved in the current study. About 100 were excluded due to various causes, including systemic disease, pregnancy, number of teeth less than 20, or patient refusal to participate in the study. The demographic data, including age and sex along with clinical periodontal parameters, are presented in Table 1: Regarding BOP, the highest value was found in the NSP group, followed by the SP group, NSH group, and SH group, with significant differences among all the groups. Concerning PPD and CAL, the SP group exhibited the highest value, followed by the NSP group, with statistically significant differences observed between the two groups.

**Figure 1:**
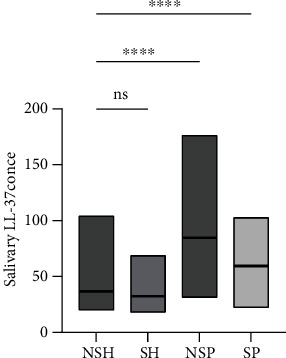
Concentration of LL-37 in saliva (ng/ml). Comparisons were done using ANOVA followed by Tukey's post hoc analysis between the nonsmoker healthy and other study groups. ^∗∗∗∗^Significant at *P* value ≤ 0.001. ns: nonsignificant; NSH: nonsmoker with healthy periodontium; SH: smoker with healthy periodontium; NSP: nonsmoker periodontitis; SP: smoker periodontitis.

The highest protein levels of salivary LL-37 were found in the NSP group, followed by the SP and NSH groups, while the SH group showed the lowest value. Statistically significant differences were observed between NSP, SP, and NSH, whereas no significant differences were found between the NSH and SH groups, as illustrated in Figure 1.

**Figure 2:**
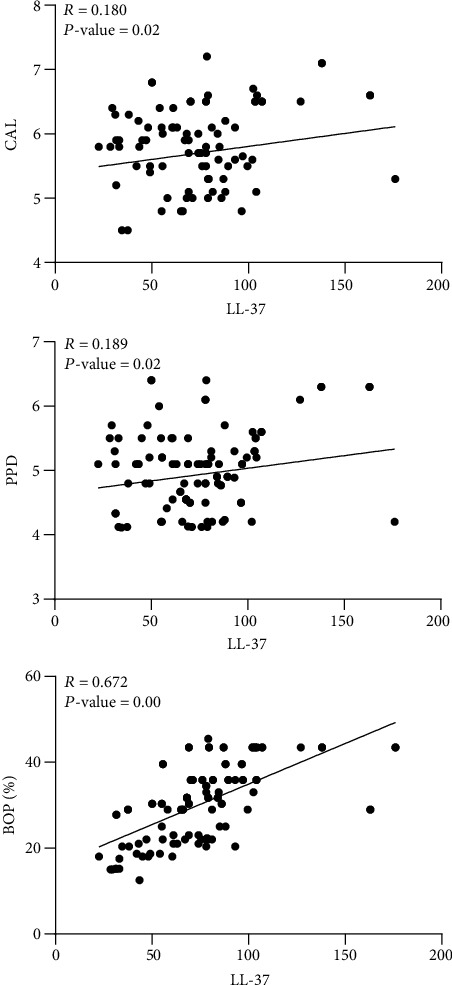
Pearson correlation between the salivary level of LL-37 and the clinical periodontal parameters. PPD: probing pocket depth; CAL: clinical attachment level; BOP%: percentage of bleeding on probing; LL-37: cathelicidin concentration in ng/ml.

Moreover, the correlation between salivary LL-37 levels and the clinical periodontal parameters revealed a statistically significant positive association with the recorded parameters (BOP, PPD, and CAL), as shown in Figure 2.

**Figure 3:**
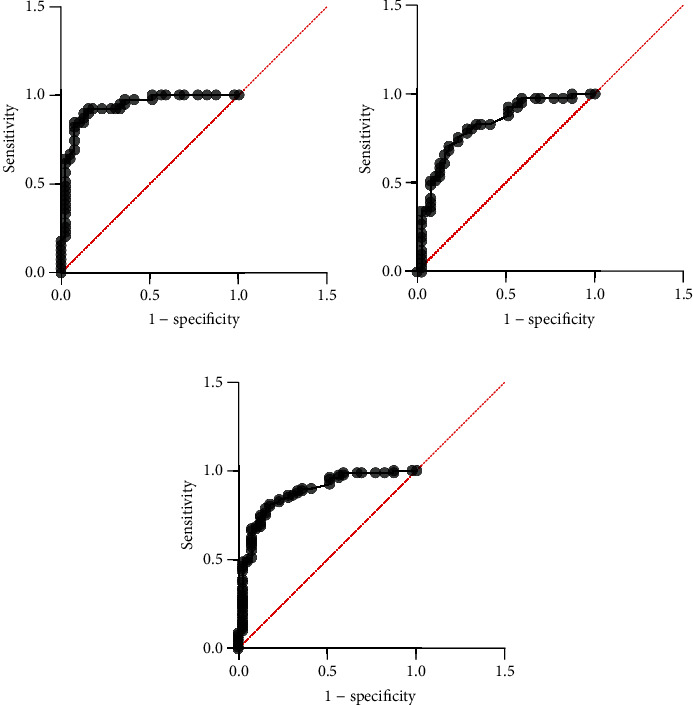
ROC curve for salivary LL-37. (a) NSH vs. NSP; (b) NSH vs. SP; (c) NSH vs. periodontitis (NSP+SP). NSH: nonsmoker healthy; NSP: nonsmoker periodontitis; SP: smoker periodontitis.

Finally, considering the diagnostic ability of salivary LL-37 to discriminate periodontal health from disease, receiver operating characteristic (ROC) was used, and the result was significant at a *P* value of 0.001, with a very good ability of salivary LL-37 as a diagnostic biomarker for periodontitis regardless of the presence or absence of smoking as shown in Table 2 and Figures 3(a)–3(c).

## 4. Discussion

Periodontitis is progressively common with a documented association with smoking as a risk factor. Furthermore, evidence of its association with many inflammatory biomarkers is emerging. In addition, clinical periodontal parameters require dental expertise to confirm the diagnosis of periodontal disease despite their limitations in detecting disease in its early stage. In contrast, identification and monitoring of disease should be possible by other clinicians and patients themselves. Consequently, the primary purpose of the present research was to assess the deleterious effect of smoking on the salivary level of LL-37 and search the diagnostic value of the current biomarker based on its reported sensitivity and specificity in relation to clinical periodontal parameters of periodontitis patient diagnosis in smoker and nonsmoker adults. Saliva is an essential diagnostic fluid due to its bioavailability and noninvasive accessibility. Besides, the composition of whole saliva comprises secretions from minor and major salivary glands, sulcular fluid, blood cells, microbes, and exfoliated epithelial cells [39]. In the recent study, the highest levels of LL-37 were observed in the NSP group, followed by the SP group (periodontitis groups), with significant differences from periodontally healthy subjects. It signifies a very good diagnostic ability to discriminate periodontitis from periodontal health regardless of the presence or absence of important risk factors such as smoking. The present result is confirmed by the direct correlation of salivary LL-37 with clinical periodontal parameters, the gold standard for periodontal disease diagnosis until now.

The activation of the immune response elicits the recruitment of neutrophils toward the site of infection, subsequently inducing the release of LL-37 from the activated neutrophils. The result of the current investigation was in accordance with a study by Türkoğlu et al. [40], which documented an upregulation in the level of expression of LL-37 mRNA in gingival tissues of patients with periodontitis. Although the current investigation measures the protein level using a different technique, ELISA, it represents a simple, noninvasive, and valid method for confirming the diagnosis. Besides, the findings of the current investigation are consistent with those of Türkoğlu et al. [41], who conducted a study on the levels of LL-37 in the gingival crevicular fluid. Their research revealed that the levels of this peptide were elevated in individuals with periodontitis who did not smoke compared to those who did smoke and had periodontitis.

The present findings contradicted the research conducted by Ribeiro et al. [42], who posited that individuals with periodontal disease exhibited reduced levels of LL-37 in their saliva. Moreover, the present study's findings were incongruent with those of Davidopoulou et al.'s investigation [43], which asserted the absence of any significant distinction in the salivary LL-37 levels between the control and periodontitis groups. The variance from the studies mentioned above could be attributed to the heterogeneity of case definition that was used at the time of diagnosis away from the new classification proposed in 2017, having an impact on the comparison of results, potentially resulting in either an overestimation or underestimation of the incidence of the disease.

The study's findings indicate that the salivary concentrations of LL-37 were comparatively reduced in the SP group as opposed to the NSP group. The literature has documented the cytotoxic impact of smoking on neutrophils. The deterioration of neutrophil chemotaxis, phagocytosis, and viability have been observed in the literature [43–46], due to smoking. This smoking-induced neutrophil dysfunction might contribute to a decrease in its release of AMPs, including LL-37. Furthermore, it has been detected that smoker individuals exhibit elevated levels of peptidyl arginine deiminase. This enzyme is responsible for the citrullination of AMPs, thereby rendering them prone to proteolysis by proteinases [47]. Consequently, this phenomenon is likely to result in a further deterioration in the salivary concentrations of LL-37.

A potential concern in the present research that was considered as a strong point is the use of salivary biomarkers in the diagnosis of periodontitis regardless of the presence or absence of important risk factors such as smoking because of the probability of systemic alterations in individuals with high-risk medical conditions and common risk factors, which may compromise the effectiveness of salivary biomarkers for diagnostic purposes. Smoking alters the balance of neutrophil functions toward a more damaging state [48], affecting the level of a biomarker used for diagnosis. Furthermore, the predetermined sample, examination with a calibrated periodontist, and blindness of the ELISA investigator and statistician all reduce the bias associated with research and strengthen the ability of the present biomarker in diagnosis.

However, the study design is observational. An optimal approach to study design would be longitudinal randomized trials to enhance the results' relevance and practicality. This study examined salivary levels of LL-37 in patients with periodontitis regardless of the severity and extent of the disease (the stage and grade of the disease), which could have an impact on the findings because previous research has demonstrated a clear relationship between the severity of periodontal inflammation and the elevation of cathelicidin levels [49–51]. Given the recent popularity of electronic cigarettes, further research is required to determine their effects.

## 5. Conclusion

Salivary LL-37 levels are significantly higher in nonsmoker periodontitis patients than in smokers, although these levels are significantly higher than control and strongly correlated with clinical periodontal parameters. This indicates that salivary LL-37 levels can be a reliable diagnostic tool for predicting periodontitis, regardless of whether the individual is a smoker. On the contrary, smoking exerts a substantial influence on diminishing these levels.

## Figures and Tables

**Table 1:** Demographic characteristics, clinical periodontal parameters, and biomarker concentration of the study groups.

Characteristics	NSH	SH	NSP	SP	*P* value
Age	42.70 ± 9.69	44.67 ± 10.02	44.65 ± 8.84	44.72 ± 8.90	0.719
Sex					
Male	31	40	35	40	—
Female	9	0	5	0	
PPD	—	—	4.81 ± 0.69	5.17 ± 0.44	0.007^∗^
CAL	—	—	5.56 ± 0.36	5.99 ± 0.05	0.001^∗^
BOP%	8.14 ± 1.30	5.59 ± 1.41^∗^^§^	35.06 ± 6.68^∗^^§^	21.37 ± 4.90^∗^^§^	0.000^∗^
LL-37 ng/ml	35.63 ± 14.07	32.54 ± 12.27	76.72 ± 17.88^∗^^§^	59.16 ± 18.90^∗^^§^	0.000^∗^

All values are presented by mean ± standard deviation except sex. Statistics were done using ANOVA for all comparisons except for PPD and CAL comparison which was done using *t*-test. NSH: nonsmoker with healthy periodontium; SH: smoker with healthy periodontium; NSP: nonsmoker periodontitis; SP: smoker periodontitis; PPD: probing pocket depth; CAL: clinical attachment level; BOP%: percentage of bleeding on probing; ^∗^Significant at *P* value < 0.05. ^∗^^§^*P* value < 0.05 ANOVA with Tukey's post hoc analysis compared with NSH.

**Table 2:** Sensitivity and specificity with the cut-off point of salivary LL-37.

LL-37	AUC	*P* value	Confidence interval	Cut-off point (ng/ml)
NSH vs. NSP	0.939	0.001	0.875 to 0.989	49.0
NSH vs. SP	0.822	0.001	0.729 to 0.915	40.5
NSH vs. periodontitis (NSP+SP)	0.876	0.001	0.808 to 0.944	47.5

AUC: area under the curve; NSH: nonsmoker healthy; NSP: nonsmoker periodontitis; SP: smoker periodontitis.

## Data Availability

On request, the data used in this investigation are available.
